# Environmental Responsibility, Social Responsibility, and Governance from the Perspective of Auditors

**DOI:** 10.3390/ijerph191912181

**Published:** 2022-09-26

**Authors:** Jaehong Lee, Suyon Kim, Eunsoo Kim

**Affiliations:** 1 Major in Accounting/Taxation, Division of Business Administration, Kyonggi University, Suwon 16227, Korea; 2 YSB Center for Global ESG and Business Ethics, Yonsei-ro 50, Seoul 03722, Korea; 3 Department of Global Business Administration, Sangmyung University, Seoul 03016, Korea

**Keywords:** ESG performance, audit hours, audit effort, managers’ ability, audit hours by rank

## Abstract

This study examined the relationship between ESGs and the audit hours of 3010 Korean stock-listed firms from 2014 to 2019. The Korean Corporate Governance Service assigns ratings to each firm based on ESG performance. We discover that auditors exert more effort auditing companies that prioritize ESGs. Additionally, we evaluated whether the CEO’s competency level influences the relationship between ESGs and the total amount of time spent auditing the company. We observed that auditors spend less time auditing companies with highly competent CEOs. We disaggregated the total number of audit hours by rank and verified each audit hour completed by partners, CPAs, as well as staff. Based on our research, we conclude that a firm’s ESG increases audit complexity, and increases the amount of effort exerted in the auditing process.

## 1. Introduction

Throughout the previous decades, consumer expectations have shifted. For instance, the majority of customers feel that companies should simultaneously work to better the environment and society while pursuing their commercial objectives [1]. ESG is an expanded notion of corporate social responsibility (CSR) that examines the sustainability of a company’s management by examining its environmentally friendly, socially responsible, and transparent corporate governance [2]. Large US companies spend roughly half of their annual budgets on corporate social responsibility [3]. BlackRock, one of the major asset management businesses in the world, claims that the worldwide investment index addressing ESG has quadrupled from $21.4 trillion in 2014 to $40.1 trillion in 2020. At the same time, South Korea’s National Pension Service has issued a directive that it would invest half of its entire assets in high ESG accomplishment enterprises by 2022. Notably, the onset of the COVID-19 pandemic has significantly increased environmental and social responsibility concerns. Furthermore, as overall interest in ESG increases, governments have also taken notice and implemented policies that reflect a prioritization of ESG ratings. According to the International Accounting Standards Board (IASB), an ESG accounting guideline will be provided, which will be included in financial statements. Furthermore, the Financial Services Commission (FSC) released a roadmap for ESG disclosure, requiring enterprises with assets worth more than $2 trillion to report ESG information.

In this study, we investigate how a company’s ESG performance affects total auditing hours. In particular, we hypothesize that auditors spend a greater amount of time with companies with ESG data as a consequence of the increased complexity of a firm’s operational environment and financial reports [4]. In other words, as a result of the increased complexity of ESG accounting and reporting, auditors will need to devote more time to these businesses than those with little or no engagement in ESGs [5].

Then, we examined how the CEO’s competency level affected the relationship between ESG and total auditing hours. The CEO of a firm is primarily responsible for developing strategy in the corporate sector. In addition, as a key decision maker, the manager is accountable for making charitable investments, such as influencing CSR-related practices [6]. Managers with high competence can effectively allocate limited resources [7] and make decisions that result in better outcomes [8]. Furthermore, highly competent managers are required to understand their financial reporting procedures [9], develop a good internal control system [10], properly anticipate profits [11], and minimize the cost of debt [12]. Auditors are required to conduct an environmental analysis of the companies they examine to fulfill their responsibility of providing reasonable assurance that the financial statements have been produced in compliance with generally accepted accounting principles (GAAP). Therefore, auditors will perceive the firms as low in risk if the firms have highly able management, which will result in a reduction in the number of audit hours required.

As part of additional tests, we investigated the connection between ESG and the total auditing hours by the auditor’s rank (partner, certified public account, and staff members); Korea is the only country that discloses auditor rank information in the audit report. After manually reviewing each audit report and collecting data in this way, we investigated the relationship between ESG and audit hours by rank. According to the findings of our investigation, ESG activities led to an increase in auditing efforts by partners, CPAs, and staff members, respectively.

Our research adds to the existing body of literature on ESG and is distinguished from other previous studies in a few notable ways. First and foremost, this is the first time the implications of ESGs have been investigated from the perspective of auditors. Despite the fact that significant research has been conducted to investigate the impact of ESG, this has primarily focused on financial performance. With this study, we examined the direct relationship between ESG factors and total auditing hours. In addition, by assessing audit hours by rank, we are able to determine that the audit hours of partners, CPAs, and staff increase in correlation to ESG activities.

The rest of the paper is organized as follows. Section 2 contains a review of the relevant literature and an explanation of our hypotheses. Section 3 outlines the data we utilized in this investigation. Section 4 reveals our findings and provides some commentary. Section 5 highlights our conclusions.

## 2. Literature and Hypothesis Development

### 2.1. Environment, Social, and Governance, and Audit Complexity

Sustainable development (SD) is strongly prioritized nowadays, and we can see this in policies such as the 2030 Agenda for Sustainable Development; these primary sustainable development goals (SDGs) address how SD progress can be measured. The concept of CSR, which was formed in response to the challenge of global sustainability, is one of the most significant actors in the promotion of SD [13]. Because of the significance of CSR, other organizations have begun implementing various strategies to promote CSR within their organizations.

For example, the B Corporation certification now incorporates societal and environmental concerns when evaluating a company’s overall business model and strategy. Also, compared to simple charitable donations in the past, B corporation certification now involves a more extensive set of requirements. Also, ESG, which is an acronym for Environmental, Social, and Governance, is emphasized.

ESG, which is a non-financial metric, is perceived as the evaluation indicator that investors utilize when making investment decisions [14] and is considered a key to investment success [15]. The demands for ESG information by investors and information intermediaries are increasing as a result of their efforts to apply this information to their valuation models [16]. In other words, a firm’s participation in ESG is recognized as an indicator of positive financial performance in the future. Similarly, the B Corporation certification, which evaluates the total company operation and is positively associated with firm performance, promotes transparency as well as sustainability [17].

Previous studies corroborate the hypothesis that firms with higher ESG ratings are more productive and financially secure because of their ability to generate more value [18]. Jeong and Shim (2019) [19] highlight the relationship between corporate social activities and a positive market reaction. Kim and Kim (2018) [20] suggest that ESG activities have a comparatively positive effect on a firm’s sustainable development compared to firms that do not partake in ESG activities. Thus, the degree of revealing that firms are dedicated to ESG is an effective mechanism to secure stakeholders’ interests and is crucial in their decision-making [21]. The information that firms disclose about their ESG activities can help improve the transparency of their financial statements and enable them to obtain further access to financial resources [22,23]. Also, revealing environmental information reduces the overall information asymmetry embedded between managers and outside stakeholders [24]. This is valuable information for stakeholders that raises awareness of the firm’s ethics; the firm’s CSR may even lead to accounting transparency by influencing the management’s attitude towards financial reporting.

The existing literature has primarily focused on the relationship between ESG and economic outcomes. However, in this study, we viewed ESG activities from the perspective of the audit. A firm’s participation in ESG gives rise to complex financial issues and requires additional accounting disclosure. The International Sustainability Standards Board mandates corporations to comply with sustainability disclosure standards, which may be onerous for a business. Furthermore, the ESG disclosure needs to comply with specific criteria; for instance, the disclosure must prove that the scenario analysis assumptions are valid [4]. Also, as Owen et al. (2001) [25] emphasized in their study, social and ethical accounting auditing and reporting involve the development of standards to create fundamental principles to assure both the quality of external reporting and the resilience of the underlying management system involved. Thus, the process of preparing ESG disclosures entails additional work that must be completed. Collectively, participating in ESG activities increases audit complexity, as investments in ESG activities trigger complex economic activities that increase the volume and variety of the accounting information that is processed.

Hay et al. (2006) [26] detail the most common aspects that lead to a complex audit: foreign subsidiaries, percentage of international assets, and industry characteristics. What these elements have in common are the metrics of complexity underpinning the auditee’s operating environment. Bonner (1994) [27] supports that the complexity of an audit stems from the various tasks that the auditor must complete to perform their duties effectively. Pirson and Turnbull (2018) [28] define the complexity encompassing financial and non-financial goals. Compared to simple donations and services in the past, ESG calls for a specific action within society. For example, for the E in ESG, which stands for environment, a revaluation of eco-friendly financing for aspects such as green bonds and assets exposed to climate risk should be factored in. For the banks whose lending portfolio is heavily exposed to carbon-intensive industries, credit risk increases when strict regulations are enforced to reduce greenhouse gas emissions. For the S category, securing adequate wages for workers should be considered (Korea Corporate Governance Service). At the same time, ESG incorporates issues related to sustainability such as environment-related opportunities, crises, and countermeasures; at the same time, sustainability also includes aspects such as efforts to improve social issues such as labor-management relations. High scores for ESG activities are achieved through positive investments in ESG, as McWilliams et al. (2006) [29] and Tsoutsora (2004) [30] suggest, which can lead to complex financial reporting issues and the need for additional accounting disclosure. 

### 2.2. Audit Risk and Audit Hours

An auditor is a person who carries out an audit, according to ISO 9001:2015 [31]. As auditors perform an audit, they are expected to have an adequate level of understanding and knowledge of auditees to verify that the financial statements are in conformity with the generally accepted accounting principles and to discover fraudulent accounting. ISO 19011:2018 offers a formal framework for professional auditors and focuses on processes that are used to acquire evidence and evaluate it objectively to assess the degree to which the audit criteria are satisfied [32]. For instance, auditors need to identify changing environments and develop a framework to elaborate the audit procedures based on ISO 19011:2018.

At the same time, when auditors plan for an audit, according to the audit risk model, auditors should determine the desirable level of detection risk that they can reasonably assess to avoid material misstatement [33]. The audit risk model is a joint probability of risk of material misstatement in the financial statement and detection risk. The risk of material misstatement in the financial statements exists due to the auditee’s inherent characteristics and its operating system. However, detection risk is under auditors’ control and they can adjust the audit risk to an acceptable or desirable level. For instance, when the auditors assess a high chance of material misstatement, they should hold the detection risk at a lower level to maintain an acceptable level of risk. 

International Standard on Auditing (ISA) 330 suggests that auditors increase their efforts when conducting audits in response to increased risks [34]. Audit efforts are related to the possibility of uncovering corporate issues [35], and audit hours will be the most relevant factor. There is prior literature measuring audit efforts based on audit fees; however, audit fees are embedded with various factors such as the risk premium, legal liability costs, and the external and internal environment of the audit market [36,37]. Thus, we focus on the audit hours as a direct measurement of audit efforts [38].

Studies on the determinants of audit hours have shown that auditors assess corporate complexity and inherent risk to determine audit efforts [39,40]. Previous studies on audit hours mainly focused on understanding the factors that determine audit hours. The variables such as total assets of the auditee, listing status, the ratio of accounts receivables and inventories in total assets, debt ratio, auditor size, and inherent risks, are the main determinants of audit hour increases [40,41,42].

Bell et al. (2001) [43] confirm that high audit hours positively correlate with high audit risk, implying that increased audit hours offset the high audit risk. Ji and Moon (2006) [44] regard reporting loss and the uncertainty of going concern as business failure. They report that firms with litigation risk due to business failure show high audit hours. Woo and Lee (2009) [45] consider the payment guarantee, equivalent to contingent liabilities, as a factor that increases audit risk, which in turn, increases audit hours. According to their analysis, it was confirmed that higher audit hours are required to have more payment guarantees provided by the auditee. In addition, the result suggests that the higher the debt ratio, the significantly longer the audit hours. Ma and Kwon (2010) [46] show the negative relationship between abnormal audit hours and income from the prior period’s error corrections, implying that the quality of audit and financial reporting improves as audit hours increase. Park et al. (2010) [47] investigate increases in total auditing hours. The result indicates that auditors exert more effort when facing audit risk, but it does not lead to higher audit fees in a competitive audit market. Overall, in the process of understanding the auditee’s objectives and strategies, auditors dedicate more time to firms with high risk.

### 2.3. Hypothesis Development

ESG refers to non-financial information that is nevertheless a crucial component of a company’s continued existence. Distinguished from simple donations made by companies, ESG is comprised of corporate policies that will be acted upon that are a critical aspect of a company’s management and a metric that is closely paid attention to and followed by investors.

Given that ESG is derived from CSR, we follow CSR’s line of reasoning as it pertains to how ESG impacts the company overall. For instance, according to Waddock and Graves (1998) [48], actively participating in CSR leads to greater financial performance, increased employee satisfaction, and increased business value. In addition to this, engaging in ESG allows for the monitoring of management, which helps decrease the risk of bankruptcy, agency expenses, and information asymmetry. Choi and Moon (2013) [49] contend that companies that engage in CSR activities have lower levels of earnings management and greater levels of earnings persistence compared to companies that do not engage in CSR activities.

Regardless of the beneficial effects, participating in ESG includes complex operating, accounting, and disclosure procedures [28], owing to the increased volume of data in the course of corporate operations [50]. Shin and An (2011) [51] investigated the association between corporate ethical management and auditing factors and found that firms focusing on ethical management are operated in a complicated manner. Pirson and Turnbuall (2018) [28] demonstrated that participation in ESG also leads to an increase in the procedures that organizations go through, which in turn increases the volume and diversity of information that an auditor is required to deal with. Since firms have the same end goal as various stakeholders to maximize profits, starting or continuing to engage in ESG builds stronger relationships with a wide range of stakeholders [52]; this further complicates the accounting process.

During the auditing process, the auditors must obtain an understanding of the company and its environment to identify events, business conditions, and activities that may have a significant impact on the risk of material misstatement [53]. Therefore, increased complexity as a consequence of ESG elevates the cognitive demands placed on auditors, which has a positive effect on the auditor’s ability to carry out the audits in an appropriate manner [26]. For instance, the high level of complexity caused by an auditee’s business environment requires the evaluation of any potential errors. The International Standard on Auditing recommends that auditors gather enough evidence to provide a reasonable level of certainty about the financial statements of the auditee [54]. In other words, if the firm carries a higher audit risk as a result of any additional or otherwise complicated tasks, the auditors will adjust the nature, timing, and extent of audit procedures, which in turn requires more effort and hours. Therefore, we set up the first hypothesis.

**Hypothesis** **1:** *There is a positive relationship between the level of ESG activities and audit hours.*

Recently, academic research has focused on the role of the top manager in understanding CSR [4]. According to the upper echelons theory, managers make strategic choices and have risk preferences depending on their psychological and cognitive characteristics [55]. Therefore, to investigate the manager’s substantial impact on ESG, the effects of cognitive and psychological characteristics that are directly connected to the manager’s decision-making capabilities should be empirically verified.

At the same time, the ISO 31000 standard provides a more comprehensive and strategic perspective to managers by outlining the ideas and practices employed in risk management. The ISO 31000:2018—Risk Management standard emphasizes the development of strategies, the accomplishment of goals, and the making of well-informed decisions to manage the effects of uncertainty on the firm’s objectives [56]. In other words, ISO 31000:2018 assists the manager in making decisions and achieving strategic goals [57].

Previous studies have shown that managerial characteristics influence a firm’s economic outcome, such as financing, operating, investment, and business practices [58,59,60]. Demerjian et al. (2012) [8] suggest that highly competent managers are likely to report precise and useful information since they understand the firms’ macroeconomics. Ban and Jeong (2018) [61] examine the impact of the managers’ ability as applied to accounting fraud, using the prediction model of accounting fraud risk, the F-score. The analysis result suggests that the manager’s competency lowers the F-score, leading to a low accounting fraud risk. Andreou et al. (2013) [62] confirm that highly competent managers make bold investments, report high performance, and reduce information asymmetry. At the same time, managerial ability plays a significant role in accounting procedures and their quality as well [63,64]. Krishnan and Wang (2015) [65] examined the impact of managers’ competence on audit fees and going concern statements. The result of the empirical analysis shows that the audit fee decreases as the competency of the manager increases. It is interpreted that the competency of the manager’s ability can reduce the likelihood of the company receiving a low possibility of going concern opinion. Specifically, auditors who have a strong preference in favor of competent managers will also have a positive prejudice that impacts the audit process [29,66]. Given the theories of a manager’s impact on a firm’s overall financial outcomes, managerial ability is one of the most important intangible assets that should be considered [8].

The auditors express the audit opinion based on whether the financial statements of companies comply with generally accepted accounting principles. The audit risk perceived by auditors varies depending on the adequacy of the financial statements. In other words, the higher the quality of accounting information, the easier it is for the auditor to judge the appropriateness of financial statements, all of which will affect audit efforts. The financial statements prepared by highly competent managers are expected to be transparent in delivering the financial messages.

Therefore, auditors need to consider the impact of tone at the top due to its prevalent impact on the auditee’s financial reporting processes [67,68]. Tone at the top affects accrual quality [69], cost of equity [70], and the forecasting by equity analysts [71]. Auditors have preferences for certain types of managers. Specifically, auditors who have a strong preference in favor of competent managers will also have a positive prejudice that impacts the audit process [72].

Based on this reasoning, firms that participate in ESG activities are perceived as more complicated by auditors than those firms that do not. Though ESG firms generally have more data [50], as well as more complex operating and business processes [28], auditors may recognize firms with competent managers as transparent and reliable in financial accounting and procedures. Therefore, the risks perceived by the auditors are expected to vary depending on the adequacy of financial statements. As both the quality and quantity of accounting information increase, it becomes easier for auditors to judge the appropriateness of financial statements, making disparate audit efforts. With this background, we set up the second hypothesis.

**Hypothesis** **2:** *Highly competent managers positively affect the relationship between the level of ESG and audit hours.*

## 3. Research Design and Sample Description

### 3.1. Research Model

#### 3.1.1. Measuring Manager’s Ability

According to Demerjian et al. (2012) [8], managerial ability is measured by how efficiently a firm generates revenue with given resources. Highly competent managers are more likely to generate higher revenue using limited resources than those with less competency. In this study, the manager’s ability is measured as suggested by Demerjian et al. (2012) [8]. Demerjian et al. (2012) [8] suggest a two-step method for quantifying managers’ ability using relative firm efficiency. In the first step, Data Envelope Analysis (DEA) is used to calculate the manager’s competency (Max), and efficiency in terms of sales relative to the inputs within the same industry. Cost of goods sold (Cogs), plant, property, and equipment (Ppe), selling, general, and administrative expenses (Sga), and intangible assets (Intan) are the input variables, and sales (Sales) the output variable, used for assessment. The following Equation (1) is used for measuring a firm’s relative efficiency.

(1) $${\mathrm{Max}}_{v}\theta =\frac{\mathrm{Sales}}{v_{1}\mathrm{Cogs}+v_{2}\mathrm{Sga}+v_{3}\mathrm{Ppe}+v_{4}\mathrm{Intan}}$$

where, Sales = firm’s sales; Cogs = cost of goods sold; Sga = selling, general, and administrative expenses; Ppe = tangible assets—land—construction in process; and Intan = intangible assets.

The second stage is to perform a Tobit regression analysis, which incorporates firm characteristic elements that impact managers’ abilities, such as firm size, market share, free cash flow, company age, number of business divisions, and foreign exchange-related variables. Managerial competence is defined as the residual that is derived by subtracting the value measured using the estimated coefficient value from the efficiency score measured by Equation (1). This process yields the value measured using the estimated coefficient value.

(2) $$\mathrm{Firm}\;\mathrm{Efficiency}=\alpha_{0}+\alpha_{1}\mathrm{Ta}+\alpha_{2}\mathrm{Ms}+\alpha_{3}\mathrm{Fcf}+\alpha_{4}\mathrm{Age}+\alpha_{5}\mathrm{Bs}+\alpha_{6}\mathrm{Fc}+\mathrm{Yr}+\varepsilon$$

where, Firm Efficiency = efficiency score measured in Equation (1); Ta = ln(total assets); Ms = sales/total revenue of firms in the industry; Fcf = 1, if Free cash flow is greater than 0, and 0 otherwise; Age = ln(firm year after listing); Bs = ln(number of business segments); and Fc = (foreign currency translation gain and loss + profit and loss on exchange)/sales.

#### 3.1.2. Testing Hypothesis

To examine the first hypothesis, whether the level of ESG affects audit hours, the following regression model is applied.

(3) $$\mathrm{Ah}=\alpha_{0}+\beta_{1}\mathrm{Esg}+\beta_{2}\mathrm{Size}+\beta_{3}\mathrm{Lev}+\beta_{4}\mathrm{Roa}+\beta_{5}\mathrm{Da}+\beta_{6}\mathrm{Mo}+\beta_{7}\mathrm{Fo}+\beta_{8}\mathrm{Beta}+\beta_{9}\mathrm{Vol}+\beta_{10}\mathrm{Big}4+\mathrm{Ind}+\mathrm{Yr}+\varepsilon$$

where, Ah = log (audit hours); Esg = log (the scores of ESG); Size = log (total assets); Lev = total liabilities/total assets; Roa = net income/total assets; Da = discretionary accruals measured in accordance with Kothari et al. (2005); Mo = percentage of shares held by majority investors; Fo = percentage of shares held by foreign investors; Beta = systematic risk; Vol = volatility of stock; Big4 = 1, if the firm is audited by a Big4 audit firm, and 0 otherwise; Ind = industry indicators; and Yr = year indicators.

The dependent variable is audit hours (Ah) in Equation (3), and it measures the total efforts of auditors shown in the auditors’ report. Esg is the score of the ESG activities, which is proprietary data acquired by the Korea Corporate Governance Service (KCGS). The control variables are referenced to the prior research of Leventis and Caramanis (2005) [73] and Park and Jeon (2012) [74]. Size is measured as the natural logarithm of the total assets. Lev is the value from total liabilities divided by total assets. Da is calculated by Kothari et al. (2005) [75], described in Equation (4).

(4) $$\frac{\mathrm{Ta}}{A}=\alpha_{0}\frac{1}{A}+\beta_{1}\frac{\left(S-\mathrm{Ar}\right)}{A}+\beta_{2}\frac{\mathrm{Ppe}}{A}+\beta_{3}\mathrm{Roa}+\varepsilon$$

where, Ta = net income—cash flow from operations; S = sales revenue; Ar = accounts receivables; Ppe = plant, property, and equipment; Roa = net income/total assets; and A = total assets.

Equation (4) is a cross-sectional model to forecast the equation for discretionary accruals. It bases its model on the industry code and the final sample includes firms with more than 15 firm-years data.

Equation (5) is the modified regression of Equation (3) to test the second hypothesis, examining whether managers’ ability moderates the relationship between ESG and audit hours. The interaction term, Esg × Ma, is of our interest.

(5) $$\mathrm{Ah}=\alpha_{0}+\beta_{1}\mathrm{Esg}+\beta_{2}\mathrm{Ma}+\beta_{3}\mathrm{Esg}\times \mathrm{Ma}+\beta_{4}\mathrm{Size}+\beta_{5}\mathrm{Lev}+\beta_{6}\mathrm{Roa}+\beta_{7}\mathrm{Da}+\beta_{8}\mathrm{Mo}\;+\beta_{9}\mathrm{Fo}+\beta_{10}\mathrm{Beta}+\beta_{11}\mathrm{Vol}+\beta_{12}\mathrm{Big}4+\mathrm{Ind}+\mathrm{Yr}+\varepsilon$$

### 3.2. Data Selection

Table 1 displays the data selection process to choose firms used in this study. The final data includes 3010 December year-end firms from 2014 to 2019. The firms are listed on the Korea Stock Exchange (KSE) and Korean Securities Dealers Automated Quotation (KOSDAQ). The firms subject to analysis have data on ESG and audit hours. Data on ESG are purchased from KCGS, the sole provider in South Korea. The ESG assessment model of KCGS is a unique evaluation model that has been designed not only following international standards such as the OECD corporate governance structure principles and ISO26000, but it also properly reflects the local legal system and business environment. Total audit hours are disclosed in the business and audit report, as well as the audit hours by rank, which requires manual collection. The financial data of control variables are collected from the FnGuide database. Due to the inconsistency that would result, financial institutions are not included. The companies that do not have the financial data are removed. To reduce the impact of outliers, the top and bottom 1% of control variables are winsorized.

## 4. Empirical Results

### 4.1. Descriptive Statistics

Table 2 shows the descriptive statistics of the main variables used in this study. The average of the dependent variable, audit hours, is 2.034. The mean (median) value of Esg is 3.335 (3.332).

Table 3 shows the correlation of the main variables in this study. The correlation between Esg and Ah is positive. We proceed with multivariate analysis considering the effect of other variables, since the result of the simple correlation is limited to conclude the result.

### 4.2. Main Findings and Discussion

Table 4 shows the result of the multivariate analysis of the relationship between Esg and audit hours, supporting the first hypothesis. The coefficient of ESG is 0.446, and is statistically significant at a 1% level, after controlling for other variables that may affect the audit hours. Based on the findings, it can be inferred that the auditors spend more time on the companies that are actively engaged in ESG. One possible interpretation of this finding is that auditors see businesses’ participation in ESG as requiring difficult or extra processes [28]. In other words, auditors view organizations’ active engagement in ESG as a high-risk factor that necessitates greater effort [26], and longer hours are spent on the auditing process. Among the control variables, Size, the measurement for firm size, shows a negative coefficient and is statistically significant. It means that as the firm size grows, the effectiveness of external audits will increase due to the high monitoring function for management. Therefore, the firm size shows a negative relationship with audit hours. Big4, an indicator of whether the firms are audited by a Big4 audit firm, shows negative value. In comparison to non-Big4, a Big4 firm has established a structured audit business, a specialized internal training program, and great audit abilities, allowing it to perform effective and efficient audits [76]. As a result, audit hour is shortened.

Table 5 is the result of the multivariate test of the second hypothesis. Hypothesis 2 assessed whether the manager’s ability moderates the relationship between ESG and audit hours. The interaction variable Esg × Ma is what we were interested in observing. The coefficient of Esg × Ma is −2.048 which is significantly negative. The finding suggests that highly capable managers may mitigate the correlation between ESG and audit hours by reducing the total amount of time spent auditing. Highly capable managers have a greater propensity to report accurate and helpful information based on their comprehension of the economic environment [9]. This reduces the likelihood of engaging in fraudulent activity [61] and lessens informational disparity [8,62]. Consequently, auditors regard companies with competent management as low-risk and reduce the number of hours spent auditing, even as risk factors increased with greater participation in ESG activities. The finding is supported by the research that Krishnan and Wang (2015) [65] conducted, which found an inverse correlation between audit efforts and level of expertise.

### 4.3. Additional Analysis—The Relationship between ESG and Audit Efforts by Rank

Table 6 shows the result of additional analysis on the relationship between ESG and audit efforts, measured in audit hours by rank. The coefficients of quality control and CPA are 0.071 and 0.066, statistically significant at 5%. The coefficients of Partners and Staffs are 0.114 and 0.287, statistically significant at 1%. The result indicates that as the firms actively participate in ESG, audit efforts by partners, CPA, staff, and quality control group increase.

Auditors are a hierarchical group of experienced professionals [77]. The audit team conducts the main tasks of the audit and ensures quality control on its own. Each audit team consists of several different members, with each member having their role to complete for the audit. Partners are the most skilled and are typically expert auditors who have more than 15 years of experience [78]. Partners are responsible for managing the overall audit and delegating audit-related tasks to the engagement team [79]. Notably, auditors disaggregate audit hours by rank [41]. They report that if the client firms carry high risk, partner efforts will increase. Chartered public accountants (CPAs) are auditors with at least 5 years of experience. While the partners’ role primarily focuses on the engagement contract, planning, and reviewing of audit reports, the CPAs are in charge of verification procedures. Finally, staff has generally had less experience compared to partners or CPAs and they assist the CPAs’ work [80]. Cameran et al. (2018) suggest that the composition of human resources on auditing teams is largely determined by the auditee’s particular circumstances. Auditing groups alter the team composition of partners, CPAs, and staff according to the team members’ roles and experiences. As such, using total audit hours is useful when generalizing information on auditing time and procedures, but the information also fails to consider differences that may arise from audit hours based on rank [41]. Therefore, it is useful to examine the effect of ESG activities on audit hours by rank.

Firms involved in ESG activities yield more complex accounting and business procedures. Auditors view this activity as both complex and having risk and they adjust the detection risk to account for this by increasing the nature, timing, and extent of the audit procedures. That is, auditors adjust efforts in accordance with the level of audit complexity. Our result supports the prior research of Johnstone and Bedard (2003) [81] and Bell et al. (2001) [43] that reported that partners, who are highly qualified auditors, are strategically assigned to company risk-related matters.

## 5. Conclusions

External auditors are one of the key stakeholders in the field of accounting. Even though there have been various studies that examine the effect of CSR, there is scant previous literature assessing the CSR effect on auditors and how auditors perceive the firms involved in CSR activities. As ESG is the developed concept of CSR that emphasizes firms’ sustainable existence, we examined the relationship between the level of ESG participation and audit hours between 2014 and 2019. Auditors regard participation in ESG activities as a factor that increases the complexity of the audit process, despite the fact that such activities may provide benefits to the company. As a result, we have concluded that involvement in ESG initiatives has a positive correlation with audit effort, which in turn helps to reduce the detection risk and provides a reasonable assurance on the audit report.

However, we find that when the firm is equipped with highly competent managers, auditors perceive that a positive tone at the top serves to moderate the higher-risk situations. Based on the additional analysis, we found a positive relationship between each hour spent by partners, CPAs, and staff, and ESG activities. We confirm that, as the firm’s complexity increased, auditors spent more effort on auditing.

Our study has several contributions to the extant literature. First, Korea has adopted a new international audit standard based on a risk-based audit approach. The risk-based approach is important and is a form of auditing process used globally. This study is meaningful in analyzing the effect of ESG activities from the perspective of auditing.

Second, prior research that examined CSR from the auditing perspective primarily focused on audit fees [5]. However, this study tests the effect of ESG on audit hours, which is a direct assessment of audit efforts. To the best of our knowledge, this study is the first to assess ESG activities from the perspective of auditors. Furthermore, this is the first study to verify the auditors’ efforts by rank. Since the audit hours by rank are disclosed officially in South Korea, we were able to generalize the results, whereas previous studies were mostly based on surveys or were limited to only certain firms [40,41,82].

The policy and practice implications of this study concentrate on how auditors deal with clients’ ESG risks. The fact that auditors usually exert more effort, which relates to a higher audit quality or audit fee, is positive for investors and ESG-supporting groups. Therefore, authorities need to update auditing risk assessment standards to incorporate ESG risk. At the same time, for efficient audits, auditors should evaluate managers’ competency in audit planning.

Several variables were not considered in this study; further studies could explore the effect of these variables on the relationship between ESG and auditing efforts.

## Figures and Tables

**Table 1:** The data selection process.

Firms with ESG, Audit Hours, and December Year-End in Years 2014–2019	3942
Less:	
No financial data	932
Final observation	3010

**Table 2:** Descriptive statistics.

Variables	Mean	STD	Q1	Median	Q3
Ah	2.034	3.051	0.000	0.000	6.418
Esg	3.335	0.399	3.091	3.332	3.584
Ma	0.001	0.114	−0.055	0.005	0.076

Definition of variables: Ah = log(Audit hours); Esg = log(Esg score); Ma = managerial ability.

**Table 3:** Pearson correlation.

	(1)	(2)	(3)
(1) Ah	1.000	−0.323	−0.015
		<0.0001	0.402
(2) Esg		1.000	−0.026
			0.146
(3) Ma			1.000
		

Notes: Ah = log(Audit hours); Esg = log(Esg score); Ma = managerial ability.

**Table 4:** The relationship between ESG and audit hours.

Variables	Estimate	T−Value
Intercept	21.509	24.790 ***
Esg	0.446	9.970 ***
Size	−0.710	−21.040 ***
Lev	−1.495	−7.750 ***
Roa	−0.331	−0.770
Da	0.782	1.780 *
Mo	−0.027	−0.110
Fo	0.498	1.420
Beta	−0.043	−0.690
Vol	6.801	1.950
Big4	−2.276	−27.050 ***
Ind	Included	
Yr	Included	
Adjusted R−square	0.367	
Observations	3010	

Notes: * and *** indicate significance at the 10% and 1% levels, respectively. Definition of variables: Ah = log(audit hours); Esg = log(the scores of Esg); Size = log(total assets); Lev = total liabilities/total assets; Roa = Net income/total assets; Da = discretionary accruals measured in accordance with Kothari et al. (2005); Mo = percentage of shares held by majority investors; Fo = percentage of shares held by foreign investors; Beta = systematic risk; Vol = volatility of stock; Big4 = 1, if the firm is audited by a Big4 audit firm, 0 otherwise; Ind= industry indicators; Yr = year indicators.

**Table 5:** The effect of managers’ ability on the relationship between ESG and audit hours.

Variables	Estimate	T−Value
Intercept	23.626	22.9600 ***
Esg	0.074	0.4900
Ma	5.583	1.6100
Esg × Ma	−2.048	−1.9100 **
Size	−0.741	−15.8900 ***
Lev	−1.585	−6.2000 ***
Roa	0.247	0.4400
Da	−0.966	−1.4300
Mo	−0.080	−0.2800
Fo	0.722	1.6800 *
Beta	0.117	1.5400
Vol	4.087	1.4000
Big4	−2.243	−21.800 ***
Ind	Included	
Yr	Included	
Adjusted R−square	0.363	
Observations	3010	

Notes:*, **, and *** indicate significance at the 10%, 5%, and 1% levels, respectively. Definition of variables: Ah = log(audit hours); Ma = managerial ability from equation (2); Esg = log(the scores of Esg); Size = log(total assets); Lev = total liabilities/total assets; Roa = Net income/total assets; Da = discretionary accruals measured in accordance with Kothari et al. (2005); Mo = percentage of shares held by majority investors; Fo = percentage of shares held by foreign investors; Beta = systematic risk; Vol = volatility of stock; Big4 = 1, if the firm is audited by a Big4 audit firm, 0 otherwise; Ind= industry indicators; Yr = year indicators.

**Table 6:** The relationship between ESG and audit hours by rank.

Variables	Partners		CPA		Staff	
	Est	T−Value	Est	T−Value	Est	T−Value
Intercept	−3.817	−13.740 ***	−4.540	−22.820 ***	−8.024	−12.240 ***
Esg	0.114	2.840 ***	0.066	2.290 **	0.287	3.040 ***
Size	0.321	25.960 ***	0.414	46.940 ***	0.330	11.340 ***
Lev	0.172	2.780 ***	0.411	9.280 ***	0.556	3.810 ***
Roa	−0.249	−2.840 ***	−0.082	−1.300	0.144	0.690
Da	−0.043	−0.300	−0.040	−0.400	−0.495	−1.490
Mo	−0.351	−4.690 ***	−0.408	−7.640 ***	0.026	0.150
Fo	0.120	1.090	−0.005	−0.060	0.376	1.460
Beta	−0.001	−0.040	0.020	1.440	−0.061	−1.340
Vol	5.475	4.870 ***	2.643	3.310 ***	3.467	1.310
Big4	−0.795	−29.780 ***	0.160	8.460 ***	4.094	65.110 ***
Ind	Included		Included		Included	
Yr	Included		Included		Included	
Adj R−square	0.366		0.657		0.670	
Observations	3010		3010		3010	

Notes: **, and *** indicate significance at the 5% and 1% levels, respectively. Definition of variables: Ah = log(audit hours); Esg = log(the scores of Esg); Size = log(total assets); Lev = total liabilities/total assets; Roa = Net income/total assets; Da = discretionary accruals measured in accordance with Kothari et al. (2005); Mo = percentage of shares held by majority investors; Fo = percentage of shares held by foreign investors; Beta = systematic risk; Vol = volatility of stock; Big4 = 1, if the firm is audited by a Big4 audit firm, 0 otherwise; Ind= industry indicators; Yr = year indicators.

## Data Availability

Not applicable.
